# A Serum Circulating miRNA Signature for Short-Term Risk of Progression to Active Tuberculosis Among Household Contacts

**DOI:** 10.3389/fimmu.2018.00661

**Published:** 2018-04-13

**Authors:** Fergal J. Duffy, Ethan Thompson, Katrina Downing, Sara Suliman, Harriet Mayanja-Kizza, W. Henry Boom, Bonnie Thiel, January Weiner III, Stefan H. E. Kaufmann, Drew Dover, David L. Tabb, Hazel M. Dockrell, Tom H. M. Ottenhoff, Gerard Tromp, Thomas J. Scriba, Daniel E. Zak, Gerhard Walzl, the GC6-74 Consortium, S. H. E. Kaufmann

**Affiliations:** ^1^The Center for Infectious Disease Research, Seattle, WA, United States; ^2^South African Tuberculosis Vaccine Initiative, Division of Immunology, Department of Pathology, Institute of Infectious Disease and Molecular Medicine, University of Cape Town, Cape Town, South Africa; ^3^Department of Medicine, Makerere University, Kampala, Uganda; ^4^Department of Microbiology, Makerere University, Kampala, Uganda; ^5^Case Western Reserve University, Cleveland, OH, United States; ^6^Max Planck Institute for Infection Biology, Berlin, Germany; ^7^DST/NRF Centre of Excellence for Biomedical TB Research and MRC Centre for TB Research, Division of Molecular Biology and Human Genetics, Faculty of Medicine and Health Sciences, Stellenbosch University, Tygerberg, South Africa; ^8^Department of Immunology and Infection, London School of Hygiene and Tropical Medicine, London, United Kingdom; ^9^Department of Infectious Diseases, Leiden University Medical Center, Leiden, Netherlands

**Keywords:** tuberculosis, microRNA, household contact, biomarker, correlate of risk, machine learning

## Abstract

Biomarkers that predict who among recently *Mycobacterium tuberculosis* (MTB)-exposed individuals will progress to active tuberculosis are urgently needed. Intracellular microRNAs (miRNAs) regulate the host response to MTB and circulating miRNAs (c-miRNAs) have been developed as biomarkers for other diseases. We performed machine-learning analysis of c-miRNA measurements in the serum of adult household contacts (HHCs) of TB index cases from South Africa and Uganda and developed a c-miRNA-based signature of risk for progression to active TB. This c-miRNA-based signature significantly discriminated HHCs within 6 months of progression to active disease from HHCs that remained healthy in an independent test set [ROC area under the ROC curve (AUC) 0.74, progressors < 6 Mo to active TB and ROC AUC 0.66, up to 24 Mo to active TB], and complements the predictions of a previous cellular mRNA-based signature of TB risk.

## Introduction

Almost one-fourth of the global population carries a latent *Mycobacterium tuberculosis* (MTB) infection (1) and is at risk of progressing to active tuberculosis. Known risk factors for progression, such as co-infection with HIV and potentially age of first exposure (2) can only explain a fraction of active disease, thus novel diagnostic and prognostic tests are needed to identify those most likely to progress (3). Accurate identification of individuals likely at high risk of active TB would facilitate prophylactic treatment strategies, potentially curing the TB infection before it progresses to its highly infectious symptomatic stage. As a first step toward this objective, we recently described a blood RNA-based correlate of risk (RNA-CoR) for progression to active TB based on splice-junction abundance from 16 interferon-response genes (4). This RNA-CoR was discovered in a South African cohort of MTB latently infected adolescents and validated using samples from South African and Gambian cohorts of household contacts (HHCs) of MTB index cases. While the results for the RNA-CoR are promising, the sensitivity and specificity of the signature were limited and there is a need to determine whether performance can be augmented using alternative approaches. The predictive power of the RNA-CoR is improved for patients close to progression to active TB. This is consistent with detection of subclinical incipient TB prior to the onset of disease symptoms. Other effective biomarkers could reflect underlying long-term risk factors that predispose individuals to develop active, rather than latent, TB after an exposure event. Exploring alternatives to whole-blood mRNA expression measurements may facilitate the discovery of these factors.

MicroRNAs (miRNAs) are small, non-coding RNAs that, as part of enzymatic protein complexes, execute post-transcriptional regulation of gene expression (5). Recent studies have demonstrated important roles for specific miRNAs during MTB infection (6). Although the established functions of miRNAs are intracellular, numerous studies have detected highly stable extracellular circulating miRNAs (c-miRNAs) in blood (7). These c-miRNAs have been explored as biomarkers for infectious diseases, including TB (8).

In this study, we evaluate c-miRNAs as candidate biomarkers for risk of TB disease progression in HHCs. These analyses make use of serum samples collected from prospective HHC cohort studies carried out in South Africa and Uganda as part of the Bill and Melinda Gates Foundation-funded Grand Challenges 6-74 program (GC6-74).

## Materials and Methods

### Study Recruitment and Sampling

Within GC6-74, 1,197 HIV-negative South African HHCs of 209 index cases were enrolled between February 27, 2006 and December 14, 2010, and 499 HIV-negative Ugandan HHCs of 181 index cases were enrolled between June 1, 2006 and June 8, 2010. HHCs from Uganda were offered INH preventative treatment. For all sites, adult participants, or legal guardians of participants aged 10–17 years old, provided written or thumb-printed informed consent to participate after careful explanation of study aims and any potential risks. All sites adhered to the Declaration of Helsinki and Good Clinical Practice guidelines in treating study participants. Ethical approvals were obtained from the relevant institutional review boards, for the South African study site, the Stellenbosch University Institutional Review Board (N05/11/187), and for the Ugandan study site, the Uganda National Council for Science and Technology (MV 715), and University Hospitals Case Medical Centre (12-95-08).

Serum samples were collected from HHCs at enrollment (within 2 months of exposure) and at 6 and 18 months after enrollment if participants remained disease free. TB progressors were defined as individuals who developed intrathoracic TB within the study period based on one of the following two criteria: (1) positive TB sputum culture coupled with at least one of the following: positive chest X-ray, positive acid-fast bacilli (AFB) sputum smear, a second positive TB sputum culture from an independent sample or clinical symptoms consistent with active TB; or (2) positive AFB sputum smear coupled with a positive chest X-ray or a second positive AFB sputum smear from an independent sample. Co-incident TB cases, defined as HHC who developed TB within 3 months of exposure, were excluded from all further analyses. At study end, controls were selected from the individuals who had remained free of active TB for the 2-year study period and matched to cases by study site, sex, age (four age groups: <18, 18–25, 25–36, >36), and year of enrollment (three enrollment groups: 2006–2007, 2008, 2009–2010). Two to three matched controls were included for each progressor. Case–control assignment was performed prior to quantification of c-miRNA levels to ensure a blind case–control design. Prior to analysis, South African samples were split into discovery and validation sets; all Ugandan samples were apportioned to the validation set.

### Serum c-miRNA Profiling and Selection

Quantification of serum c-miRNA levels was performed by Exiqon Inc. (Vedbaek, Denmark) using qRT-PCR with locked-nucleic acid primers as previously described (9). Briefly, total RNA was extracted from serum using the miRCURY™ RNA isolation kit—biofluids (Exiqon, Inc., Vedbaek, Denmark) as follows. Serum/plasma was thawed on ice and centrifuged at 3,000 × *g* for 5 min in a 4°C microcentrifuge. An aliquot of 200 µL of serum/plasma per sample was transferred to a new microcentrifuge tube and 60 µL of Lysis solution BF containing 1 µg carrier-RNA per 60 µL Lysis Solution BF and RNA spike-in template mixture was added to the sample. The tube was vortexed and incubated for 3 min at room temperature, followed by addition of 20 µL Protein Precipitation solution BF. The tube was vortexed, incubated for 1 min at room temperature and centrifuged at 11,000 × *g* for 3 min. The clear supernatant was transferred to a new collection tube, and 270 µL isopropanol was added. The solutions were vortexed and transferred to a binding column. The column was incubated for 2 min at room temperature, and emptied using a vacuum-manifold. 100 µL wash solution 1 BF was added to the columns. The liquid was removed using a vacuum-manifold, and 700 µL wash solution 2 BF was added. The liquid was removed using a vacuum-manifold. 250 µL wash solution was added and the column was spun at 11.000 × g to dry the columns entirely. The dry columns were transferred to a new collection tube and 50 µL RNase free H_2_O was added directly on the membrane of the spin column. The column was incubated for 1 min at room temperature prior to centrifugation at 11,000 × *g*. The RNA was stored in a −80°C freezer.

2 µL RNA was reverse transcribed in 10 µL reactions using the miRCURY LNA™ Universal RT microRNA PCR, Polyadenylation, and cDNA synthesis kit (Exiqon, Inc., Vedbaek, Denmark). cDNA was diluted 50× and assayed in 10 µL PCR reactions according to the protocol for miRCURY LNA™ Universal RT microRNA PCR; each microRNA was assayed once by qPCR on the microRNA Ready-to-Use PCR, Pick-n-Mix using ExiLENT SYBR^®^ Green master mix. Negative controls excluding template from the reverse transcription reaction was performed and profiled like the samples. The amplification was performed in a LightCycler^®^ 480 Real-Time PCR System (Roche) in 384 well plates. The amplification curves were analyzed using the Roche LC software, both for determination of Cq (by the second derivative method) and for melting curve analysis. Two technical replicates were performed for each sample, and mean *C_t_* values for each c-miRNA in each sample, along with experimental metadata are provided in Table S1 in Supplementary Material.

An initial panel of 608 c-miRNAs were considered for analysis, based on miRNA primers suggested by Exiqon, Inc. including c-miRNAs previously suggested as potential biomarkers (Table S2 in Supplementary Material). This panel was down-selected to 164 c-miRNA (Table S2 in Supplementary Material) based on detectable expression in >80% of samples and association with progression in a subset of 40 discovery set samples. The technical replicability of each of the 164 initial candidate miRNAs was then assessed by rerunning the PCR quantification of the candidate miRNA, resulting in two technical replicates for each sample. The quality of the replicates was assessed by measuring the Pearson correlation of individual miRNAs between technical replicates. We observed a strong, non-linear relationship between miRNA expression (as measured by *C_t_*) and technical replicability. In particular, a sharp decline in replicability was observed for miRNAs with mean *C_t_* values greater than 32, indicative of low levels of c-miRNA (Figure S4 in Supplementary Material). A final panel of 47 candidate miRNAs was thus selected, comprised of miRNAs expressed at reliably detectable levels (*C_t_* < 32) in serum. PCR quantification of these 47 miRNAs was then run on all samples, including the pilot study samples.

### Normalization of PCR c-miRNA Data

As the abundance of c-miRNAs in serum is relatively low and varies across conditions, there is currently no universally accepted set of reference “housekeeping” c-miRNAs or universally accepted approach for standardizing c-miRNA profiles in order to maximize comparability across samples. To address this issue, we explicitly evaluated multiple normalization approaches within the suite of machine-learning approaches employed to generate predictive signatures. If a particular normalization strategy was strongly superior or inferior than others, this difference would be evident as increased or decreased predictive accuracy when assessed during cross-validation of the discovery set. The normalization strategies that we investigated were variants of two classes. In the first class, subsets of potential reference c-miRNAs were selected by ranking the final panel of 47 c-miRNAs by the magnitude of Spearman rank correlation between the c-miRNA and the overall sample mean of the *C_t_*s of all 47 miRNAs. The assumption behind this approach is that any universal difference in c-miRNA abundance between samples would be due to technical reasons (like smaller or less concentrated plasma aliquot) as opposed to biological reasons. The c-miRNAs with the top 1, 3, 5, 10, 20 rank correlations to the overall sample mean would be selected as reference c-miRNAs and then averaged within each sample to generate per-sample normalization constants. Alternatively, for the second class of approaches, the per-sample normalization constants were generated by taking the mean, median, or 25% trimmed-mean computed from all 47 assayed c-miRNAs. The *C_t_*s for a given sample were then normalized by subtracting the value of the normalization constant from the *C_t_* of each c-miRNA. This gave a total of eight normalized datasets: trimmed-mean, trimmed-median, 1-ref, 3-ref, 5-ref, 10-ref, 20-ref, or 47-ref (i.e., mean) normalized.

### c-miRNA Signature Development

**Figure 1:**
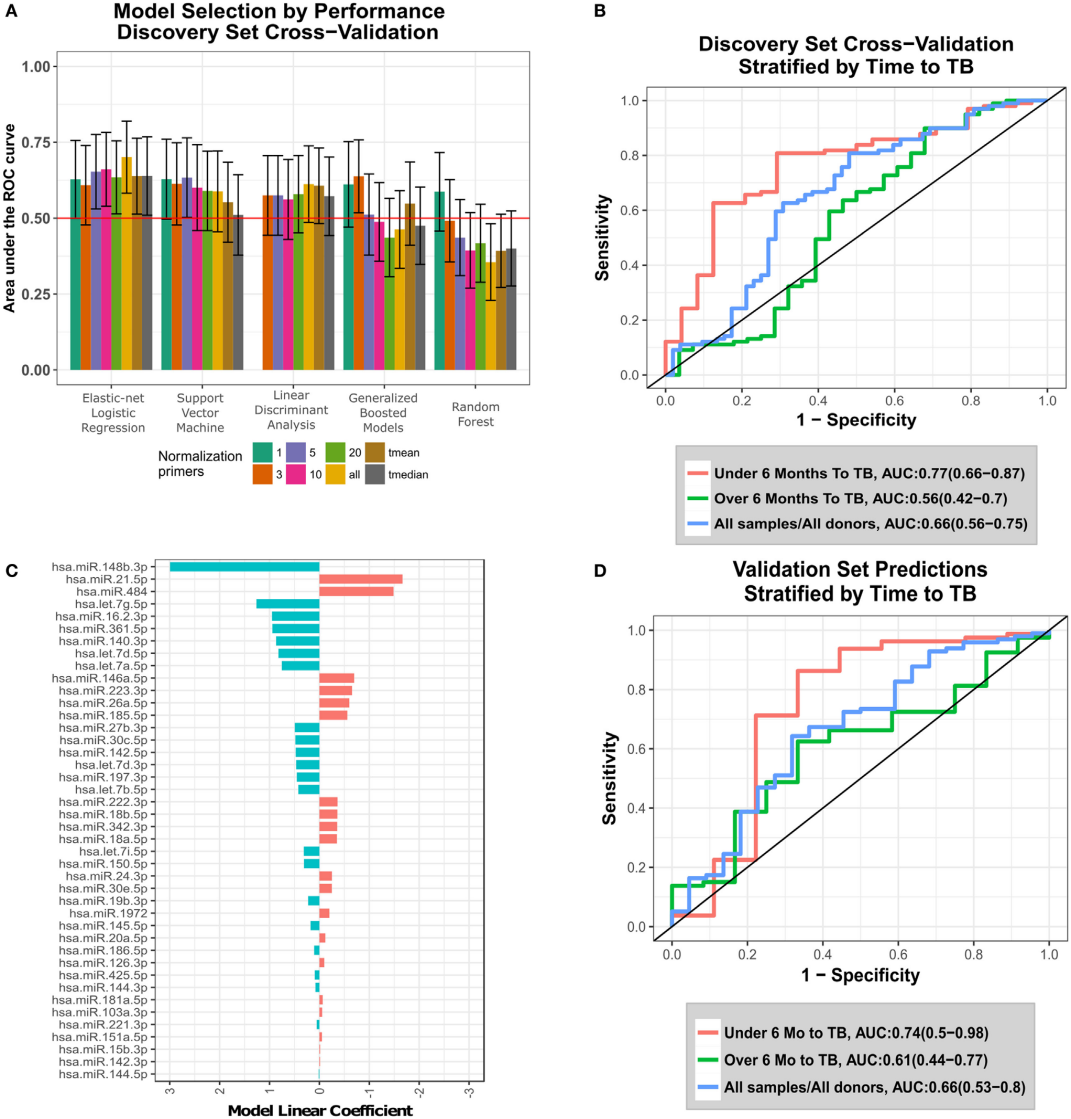
Development and validation of the circulating microRNA (c-miRNA) TB risk signature. **(A)** ROC area under the ROC curves (AUCs) from discovery set leave-one-donor-out-cross-validation (LOOCV) for five different machine-learning algorithms applied to data generated using eight different normalization approaches. Error bars represent the 95% confidence intervals. Normalization primers indicate the numbers of reference primers used to normalize the data (“all” = all 47 primers, and “*t*mean” and “*t*median” = 25% trimmed-mean or median of all primer expression, respectively). Horizontal red line indicates non-discrimination (AUC = 0.5). The machine-learning algorithms employed are indicated on the *x*-axis. **(B)** LOOCV ROC curves for the optimal algorithm (elastic-net logistic regression-all), stratified by the time between collection of the sample and TB diagnosis (time to TB). **(C)** Values of fitted linear coefficients for each c-miRNA in the final logistic regression signature. Red and blue indicate c-miRNAs detected at higher and lower levels in the serum of progressors compared with controls, respectively. **(D)** Validation set blind prediction ROC curves for the optimal TB risk signature with progressors stratified by time to TB as in **(B)**.

The predictive potential of candidate c-miRNA signatures of risk was estimated by leave-one-donor-out-cross-validation (LOOCV) of the discovery set measurements of the 47 c-miRNAs. To ensure unbiased cross-validation, all samples relating to one donor were held out, the machine-learning algorithm was fit to the remaining data, and the resulting fit used to make blind predictions on the held-out samples. This step was done for each donor, and repeated for every combination of machine-learning algorithm and normalization approach. Using the R package caret (10), a variety of machine-learning algorithms were assessed (Figure 1).

Five machine-learning algorithms were used to train predictive models on the miRNA datasets, with models trained using the R caret (10) package as an interface: Random Forest [R randomForest package (11)]; Support Vector Machine using RBF kernel [R kernlab (12) package]; Neural Networks [R nnet (13) package]; Elastic-net Logistic Regression [R glmnet (14) package]; and Linear Discriminant Analysis (13). Initial performance was assessed using LOOCV during training. During LOOCV, all samples relating to a single donor were held out and predicted on together, i.e., samples taken at differing timepoints from a single donor. In the discovery analysis, the optimal model was selected by examining LOOCV predictive performance considering only the sample most proximal to TB diagnosis.

The R pROC (15) package was used to calculate ROC curves by applying a set of thresholds to numeric predictions from predictive models to predict the progressor or control status of the samples, and then calculating the sensitivity and specificity of the predictor at each threshold. ROC curves were plotted using the R ggplot2 (16) package. Accompanying positive and negative predictive values were calculated using the model prediction threshold that maximized the sum of sensitivity and specificity.

Prediction performance, as measured by ROC statistics, was assessed using the sample for each participant that was most proximal to TB diagnosis. The combination of algorithm and normalization that maximized the area under the ROC curve (AUC) was selected to construct the final signature and was then used to make blind predictions on the validation set. *p*-Values associated with each signature were calculated using a one-tailed Mann–Whitney *U*-test comparing signature scores for cases compared with controls and were adjusted for multiple testing using the Benjamini–Hochberg algorithm. Bootstrapping was used to estimate 95% confidence intervals (CIs) of the AUC.

### Prediction Performance of Combined RNA + c-miRNA Signature

To determine whether combining the c-miRNA signature with the existing RNA-based risk signature (RNA-CoR) led to significant improvement in prediction accuracy, a χ^2^ test was performed comparing two logistic regression models: (1) *Progression* = *f(RNA-CoR* + *c-miRNA)* and (2) *Progression* = *f(RNA-CoR)*. This approach takes into account the nested nature of these models. The significance of the improvement in the combined models’ AUC was also evaluated using the highly conservative (17, 18) DeLong (19) test, which assumes the independence of the models. These analyses were performed using samples for which both RNA-CoR scores (4) and c-miRNA signatures scores were available (34 progressor samples, 79 control samples) from both the training and test sets. To conservatively estimate c-miRNA signature performance, c-miRNA scores from the cross-validation analysis were used for training set samples and from the blind prediction analysis for the test set samples. Spearman correlations between normalized RNA-CoR PCR data (4) and normalized c-miRNA data were also calculated using matching samples.

## Results

### Establishment of Study Cohorts

43 and 11 HHCs from the South African and Ugandan cohorts, respectively, progressed to active TB (“progressors”) and were matched to HHCs that had remained healthy (“controls”) during the 2-year study period (summarized in Table S3 in Supplementary Material). Tuberculin skin test (TST) measurements at enrollment found 91% of participants to have TST indurations ≥10 mm and 75% ≥15 mm, suggesting that the vast majority of HHCs have a latent TB infection. TST induration size did not differ significantly between progressors and controls (*U*-test *p* = 0.78), indicating that the TST is an ineffective predictor of TB risk in these cohorts. This ineffective prediction is unlikely to be related to false positives caused by BCG vaccination or TST cross reactivity with non-tuberculous mycobacteria (20) and the large TST indurations are more likely to reflect latent *M. tuberculosis* infection. Compared with our previous study of progression in South African adolescents with latent TB where 0.7% of individuals progressed to active TB over the course of 2 years (4), 3.6% of South African HHCs progressed to active TB.

A panel of 47 high expression, technically replicable c-miRNAs were selected from 608 candidate miRNAs. These 47 c-miRNAs were then analyzed in parallel on the discovery (151 samples) and validation (120 samples) sets.

### Generation and Validation of the c-miRNA Signature of TB Risk

To identify an optimal c-miRNA signature of risk for TB among HHCs, we evaluated five different machine-learning algorithms using eight different normalization strategies (see Materials and Methods, Figure 1A; Table S4 in Supplementary Material). The top algorithm was elastic-net logistic regression normalized by the average of all 47 c-miRNAs, which achieved a cross-validation AUC of 0.7 (95% CI: 0.58–0.82, FDR-adjusted *p* = 0.04, negative predictive value = 81%, positive predictive value = 59%) (Figure 1A). Figure 1B shows ROC curves for LOOCV results stratified by the time between sample collection and TB diagnosis [“Time To TB”, as in Ref. (4)]. Predictions for samples within 6 months of progression (AUC: 0.77, CI: 0.66–0.87, NPV = 92%, PPV = 47%) were superior compared with those at all times to progression (AUC: 0.66, CI: 0.56–0.75, NPV = 76%, PPV = 59%). Significant predictions were also observed when considering baseline samples only (AUC: 0.63, CI: 0.5–0.77, Figure S1 in Supplementary Material). The optimal final signature selected was trained on the entire discovery set (Figure 1C; Table S5 in Supplementary Material). Blind prediction of TB progression by the signature when applied to the validation set was successful (ROC AUC = 0.66, CI: 0.53−0.8, NPV = 90%, PPV = 30%) when applied to all samples; Figure 1D. Stronger performance was observed on samples under 6 months to TB (ROC AUC = 0.74, CI: 0.5−0.98, NPV = 96%, PPV = 35%), consistent with the discovery set. While the signature was not significantly predictive on the baseline validation samples, i.e., samples taken close to study enrollment (AUC: 0.55, CI: 0.32–0.77, NPV = 83%, PPV = 37%), Figure S1 in Supplementary Material, very strong significant predictive performance was seen on baseline validation set samples within 6 months of TB progression (AUC: 0.95, CI: 0.88–1, NPV = 100%, PPV = 50%), Figure S1 in Supplementary Material. These results demonstrate that a c-miRNA derived signature significantly predicts TB risk for HHCs within 6 months of progression.

### Drivers of the c-miRNA Signature of TB Risk

**Figure 2:**
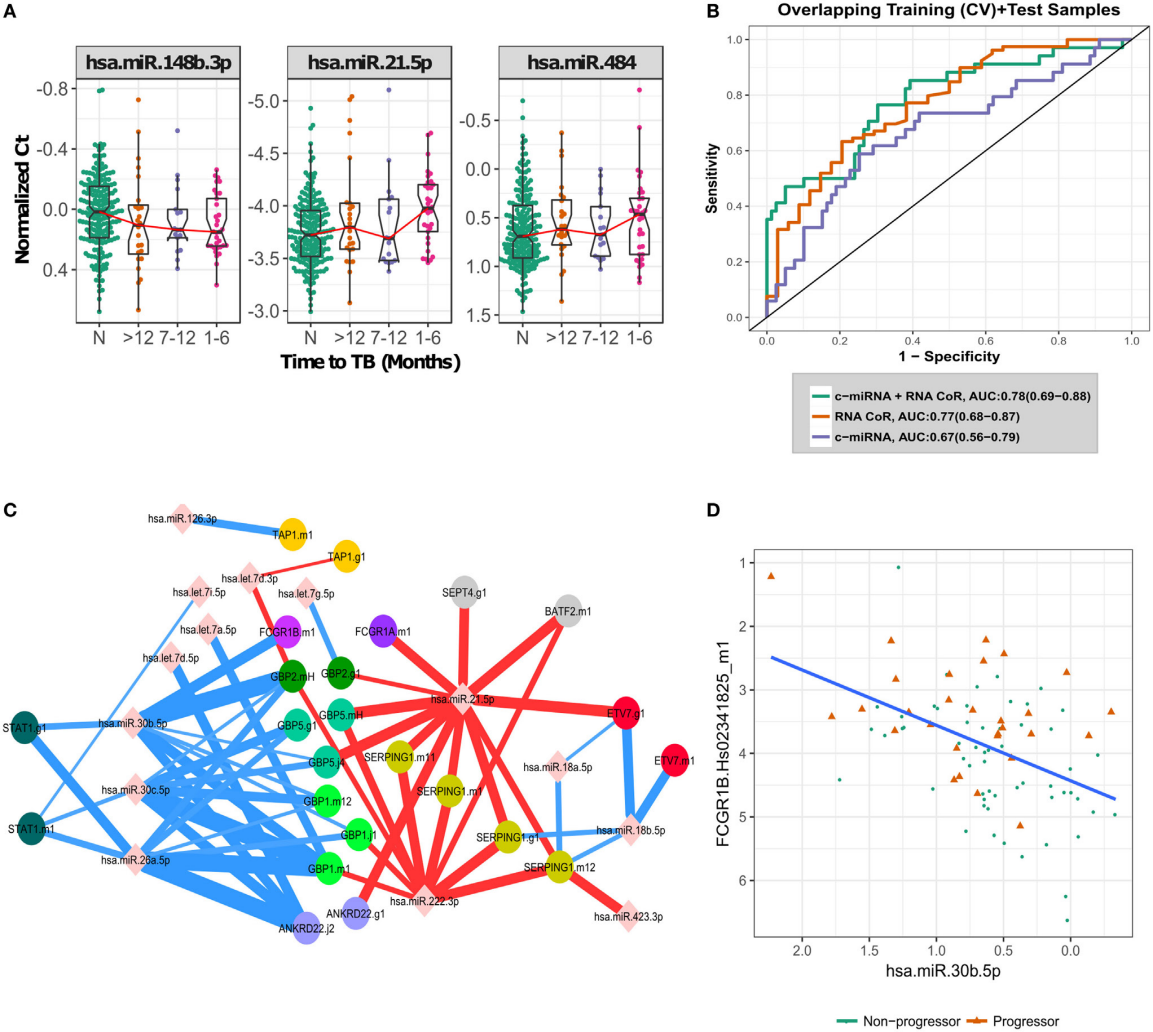
Biological signal underlying the circulating microRNA (c-miRNA) signature. **(A)** Normalized PCR *C_t_* values for the three most important c-miRNAs in the signature, with progressors stratified by time to active TB disease. “N” indicates non-progressor control samples. **(B)** ROC curves illustrating RNA-based correlate of risk (RNA-CoR) prediction score, c-miRNA (leave-one-donor-out-cross-validation + blind prediction scores for the 47 miRNA model) and combined score performance at classifying the shared set of discovery and validation samples. **(C)** Correlation network of c-miRNA and RNA-CoR gene PCR primers. c-miRNA—gene correlations calculated using Spearman’s rho with FDR < 0.05 are indicated by edges connecting miRNAs to genes. Edge thickness is proportional to significance of the correlation. Positive correlations are indicated in red, with negative correlations in blue. **(D)** Correlation between FCGR1B and miRNA hsa-miR-30b-5p, linear best fit line shown in blue.

Having validated the c-miRNA signature of TB risk, we performed a retrospective analysis to determine which c-miRNAs were the drivers of prediction accuracy. By sequentially removing c-miRNAs with the smallest model weight, retraining on the discovery set, and predicting on the validation set, we were able to identify the most parsimonious predictive signature (Figure S2 in Supplementary Material, Table S6 in Supplementary Material). Although prediction performance fluctuated stochastically with an overall decline as the signature was reduced, a three-c-miRNA signature predicted comparably to the full signature (AUC: 0.67, CI: 0.55–0.80, NPV = 78%, PPV = 64%), indicating potential for model reduction. Figure 2A shows the combined discovery and validation set expression of the three c-miRNAs. Thus, it appears signature predictions are dominated by the contribution of the three most important miRNAs.

### The c-miRNA Signature of TB Risk Complements the RNA-CoR Predictions

The c-miRNA signature of TB risk includes c-miRNAs up- and down-regulated in TB progression, in contrast with the transcriptomic RNA-CoR (4) which was composed of genes upregulated during progression. These distinct kinetics suggest that the c-miRNA and RNA-CoR signatures may contain independent information for predicting TB among HHCs. The South African samples used to validate the RNA-CoR form part of this study cohort, facilitating a direct comparison of the c-miRNA signature with the published qRT-PCR RNA-CoR measurements. A linear combination of the c-miRNA, including all 47 miRNAs, and RNA-CoR signatures shows a modest increase in predictive power, from an AUC of 0.77 (CI: 0.68–0.87, NPV = 88%, PPV = 48%) using RNA-CoR alone to 0.78 (CI: 0.69–0.88, NPV = 87%, PPV = 52%) for the combined signature (Figure 2B), and we observed wide overlap of the 95% CI between the RNA-CoR alone and the RNA-CoR + c-miRNA model. Although the AUCs of the RNA-CoR + c-miRNA did not significantly improve on the RNA-CoR when compared using the conservative DeLong test (*p* = 0.43), significant (*p* = 0.03) improvement in predictive performance was observed when the linear combination of RNA-CoR + c-miRNA was compared with RNA-CoR alone using the χ^2^ test, which takes into account the nested nature of the models. Notably, predictions were strongly improved in the high-specificity region of the ROC curve, at a specificity of 90%, where RNA-CoR shows a sensitivity of 41%, which improves to a sensitivity of 50% when the c-miRNA scores are added.

To further explore the relationship between the c-miRNA and cellular RNA expression changes, we performed a correlation analysis between the constituents of the two signatures. Figure 2C shows a network of significant (FDR < 0.05) correlations between the components of the c-miRNA and RNA-CoR signatures (Table S7 in Supplementary Material). Both positive and negative correlations between c-miRNAs and the interferon-response genes in the RNA-CoR were observed in a manner consistent with previous functional studies of the implicated RNAs (21–25) (Figure 2D). These results demonstrate that elements of the c-miRNA signature are correlated with the previously identified RNA-CoR, and that the c-miRNA signature may provide information complementary to the RNA-CoR.

## Discussion

Several previous studies have identified c-miRNAs that are differentially expressed in active TB disease (8), but to our knowledge, this is the first to have prospectively validated a c-miRNA-based signature of risk of TB in an independent cohort. The c-miRNAs comprising the signature are abundant in blood and have established roles in inflammatory and infectious conditions (21, 23–25). This signature is highly predictive of HHCs likely to progress within 6 months of testing, including tests performed close to exposure, although predictive power is diminished for more distal samples. This increase in signal close to diagnosis suggests that the c-miRNA signature is likely to be detecting an immune response to subclinical or incipient TB, prior to the development of symptomatic active disease. We observed that most progressors developed TB within 6 months of exposure (Figure S3 in Supplementary Material), suggesting that the temporal resolution of this test may be sufficient for practical application. As our analysis was limited to previously characterized c-miRNAs, we could not have identified potentially important uncharacterized c-miRNAs. Future improvements in sequencing approaches have potential to identify additional c-miRNAs that may be important in the context of TB progression.

The RNA-CoR signature has been shown to have over double the positive predictive value of an interferon-gamma release assay alone and meets the Stop TB Partnership’s performance criteria for a prognostic TB test (26). Combined with the RNA-CoR, the c-miRNA signature displays only a slight improvement in AUC vs the RNA-CoR alone. However, the predictive performance shows a strong improvement in sensitivity at high specificities, suggesting that combination of the RNA-CoR and c-miRNA signature would act as an improved “rule-in” test to identify HHCs at higher risk and likely to benefit from INH prophylaxis.

Correlating the components of the c-miRNA signature with components of the RNA-CoR signature suggest how the interferon response to TB disease may be regulated by c-miRNAs. miR-21, which is induced by mycobacteria (21), and is a marker of immune cell activation (24), was positively correlated with genes in the RNA-CoR. In contrast, miR-26a, which has been shown to suppress macrophage responsiveness to IFN-γ (23), and miR-30b, which has been shown to suppress pro-inflammatory cytokine secretion and Fc-receptor expression (25), were both negatively correlated with RNA-CoR genes, including *FCGR1B* (Figure 2D).

Recently, blood transcriptional signatures have been developed capable of evaluating TB risk (4) and effective response to TB treatment outcome (27), although the sensitivity and specificity of the risk signature is limited. Investigating alternative platforms to whole-blood transcription holds out the possibility of augmenting the performance of this initial work. The c-miRNA signature developed here demonstrates the potential of serum c-miRNAs to predict TB risk, despite being limited by a preselected pool of candidate miRNAs, and the difficulty of accurately quantifying low-abundance miRNAs in serum. In the future, the development of accurate, sensitive, and unbiased sequencing approaches for c-miRNAs would hold much promise for further improving prediction of TB risk.

## The GC6-74 Cohort Study Group

Germany: **S. H. E. Kaufmann** (GC6-74 Principal Investigator), **S. K. Parida**, **R. Golinski**, **J. Maertzdorf**, **J. Weiner III**, **M. Jacobson**, **G. McEwen** (Department of Immunology, Max Planck Institute for Infection Biology, Berlin). South Africa: **G. Walzl**, **G. Black**, **G. van der Spuy**, **K. Stanley**, **M. Kriel**, **N. Du Plessis**, **N. Nene**, **A. Loxton**, **N. N. Chegou** (DST/NRF Centre of Excellence for Biomedical TB Research and MRC Centre for TB Research, Division of Molecular Biology and Human Genetics, Stellenbosch University, Tygerberg); **S. Suliman**, **T. Scriba**, **H. Mahomed**, **M. Erasmus**, **O. Xasa**, **A. Veldsman**, **J. Hughes**, **K. Downing**, **A. Penn-Nicholson**, **H. Mulenga**, **B. Abel**, **M. Bowmaker**, **B. Kagina**, **W. Kwong C.**, **W. Hanekom** (South African Tuberculosis Vaccine Initiative, Institute of Infectious Disease and Molecular Medicine & Department of Paediatrics and Child Health, University of Cape Town, Cape Town). Netherlands: **T. H. M. Ottenhoff**, **M. R. Klein**, **M. C. Haks**, **K. L. Franken**, **A. Geluk**, **K. E. van Meijgaarden**, **S. A. Joosten** (Department of Infectious Diseases, Leiden University Medical Centre, Leiden); **D. van Baarle**, **F. Miedema** (University Medical Centre, Utrecht). USA: **W. H. Boom**, **B. Thiel** (Tuberculosis Research Unit, Department of Medicine, Case Western Reserve University School of Medicine and University Hospitals Case Medical Center, Cleveland, Ohio); **J. Sadoff**, **D. Sizemore**, **S. Ramachandran**, **L. Barker**, **M. Brennan**, **F. Weichold**, **S. Muller**, **L. Geiter** (Aeras, Rockville, MD); **G. Schoolnik**, **G. Dolganov**, **T. Van** (Department of Microbiology and Immunology, Stanford University, Stanford, California). Uganda: **H. Mayanja-Kizza**, **M. Joloba**, **S. Zalwango**, **M. Nsereko**, **B. Okwera**, **H. Kisingo** (Department of Medicine and Department of Microbiology, College of Health Sciences, Faculty of Medicine, Makerere University, Kampala). UK: **H. Dockrell**, **S. Smith**, **P. Gorak-Stolinska**, **Y.-G. Hur**, **M. Lalor**, **J.-S. Lee** (Department of Immunology and Infection, Faculty of Infectious and Tropical Diseases, London School of Hygiene & Tropical Medicine, London). Malawi: **A. C. Crampin**, **N. French**, **B. Ngwira**, **A. B. Smith**, **K. Watkins**, **L. Ambrose**, **F. Simukonda**, **H. Mvula**, **F. Chilongo**, **J. Saul**, **K. Branson** (Karonga Prevention Study, Chilumba). Ethiopia: **D. Kassa**, **A. Abebe**, **T. Mesele**, **B. Tegbaru** (Ethiopian Health & Nutrition Research Institute, Addis Ababa); **R. Howe**, **A. Mihret**, **A. Aseffa**, **Y. Bekele**, **R. Iwnetu**, **M. Tafesse**, **L. Yamuah** (Armauer Hansen Research Institute, Addis Ababa). The Gambia: **M. Ota**, **J. Sutherland**, **P. Hill**, **R. Adegbola**, **T. Corrah**, **M. Antonio**, **T. Togun**, **I. Adetifa**, **S. Donkor** (Vaccines & Immunity Theme, Medical Research Council Unit, Fajara). Denmark: **P. Andersen**, **I. Rosenkrands**, **M. Doherty**, **K. Weldingh** (Department of Infectious Disease Immunology, Statens Serum Institute, Copenhagen).

## Ethics Statement

This household contact study included participants from two African sites: South Africa and Uganda, under the Bill and Melinda Gates Grand Challenges 6-74 (GC6-74) program. All clinical sites adhered to the Declaration of Helsinki and Good Clinical Practice guidelines, and ethical approvals were obtained from institutional review boards at both sites. Ethics review committee names and protocol numbers for each are listed in Materials and Methods.

## Author Contributions

FD, ET, and DZ carried out the computational analyses and drafted the manuscript. WB, SK, HD, TO, TS, and GW conceived the experimental and study design. KD, SS, HK, BT, JW, DD, DT, GT, DZ, and GW designed, oversaw and performed subject recruitment, biological sample collection and experimental procedures. All authors contributed to writing and revising the manuscript.

## Conflict of Interest Statement

The authors declare that the research was conducted in the absence of any commercial or financial relationships that could be construed as a potential conflict of interest. The handling Editor declared a shared affiliation, though no other collaboration, with several of the authors KD, SS, and TS.
