# Supplementary material for: A Serum Circulating miRNA Signature for Short-Term Risk of Progression to Active Tuberculosis Among Household Contacts
Source: Front Immunol. 2018 Apr 13;9:661. doi: 10.3389/fimmu.2018.00661 (PMC5908968; doi:10.3389/fimmu.2018.00661)
Supplement: Supplementary file 1 [file image_1.PDF]

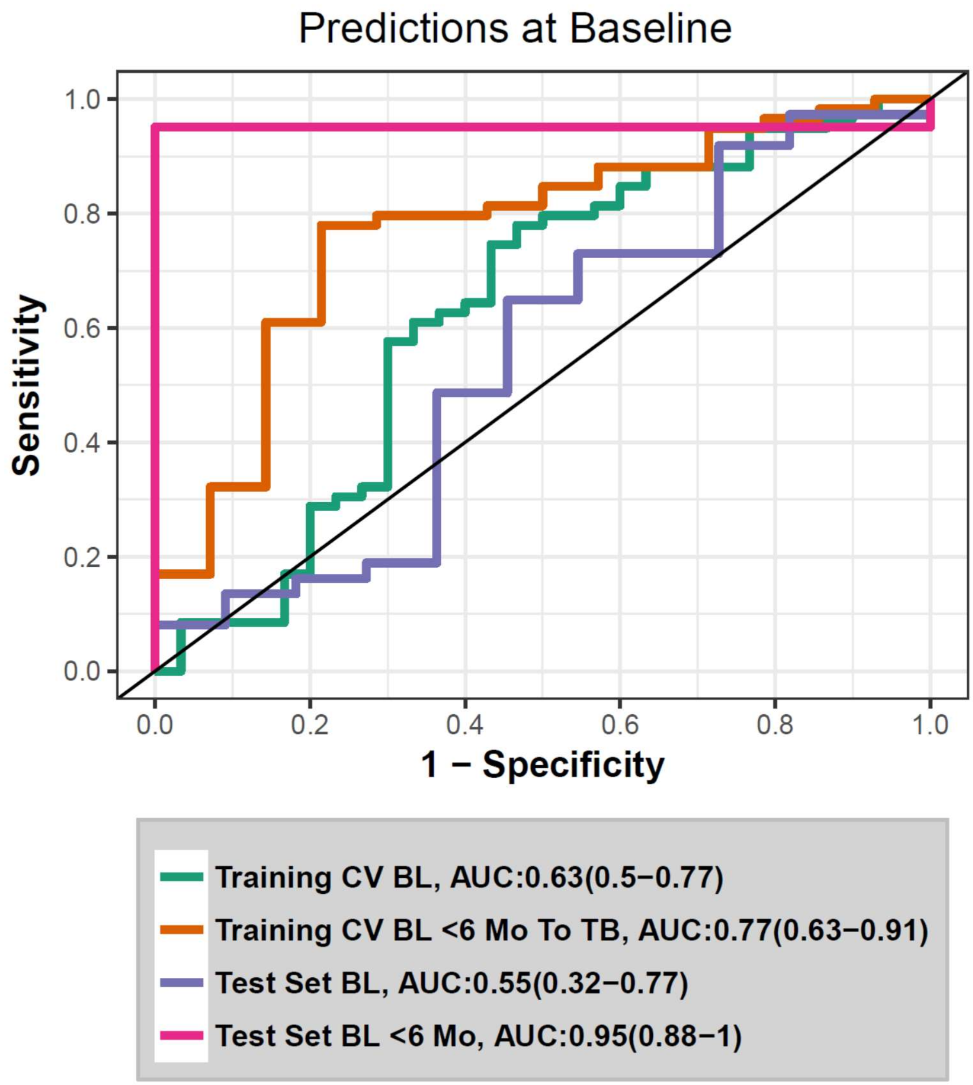

Figure S1: c-miRNA signature predictions on training and test baseline samples stratified by time to TB. ROC curves for the 47 c-miRNA signature predictions on the training and test sets considering samples taken at baseline/study enrollment only (BL). Leave-one-out CV predictions are shown for the training set, and blind prediction results for the test set. <6 Mo to TB indicates that only TB progressors within 6 months of active disease have been included.

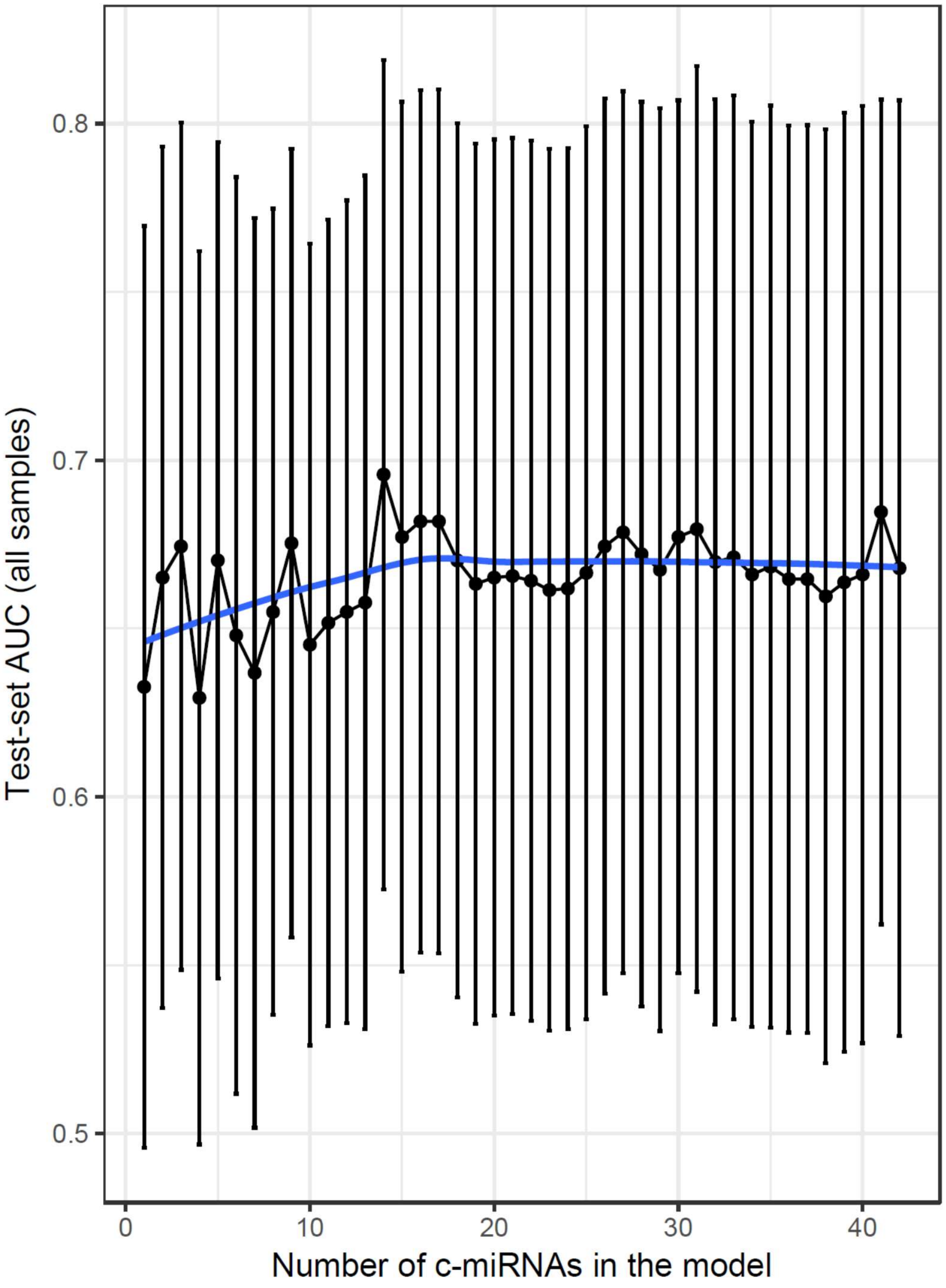

Figure S2: <u>Change in test set ROC AUC after sequential removal of c-miRNAs</u>. X-axis shows the number of c-miRNAs in the signature, with test set predictive performance in terms of ROC AUC on the Y-axis. Error bars show 95% confidence intervals around the AUC. Blue line is the smoothed best fit line determined using local polynomial regression (loess).

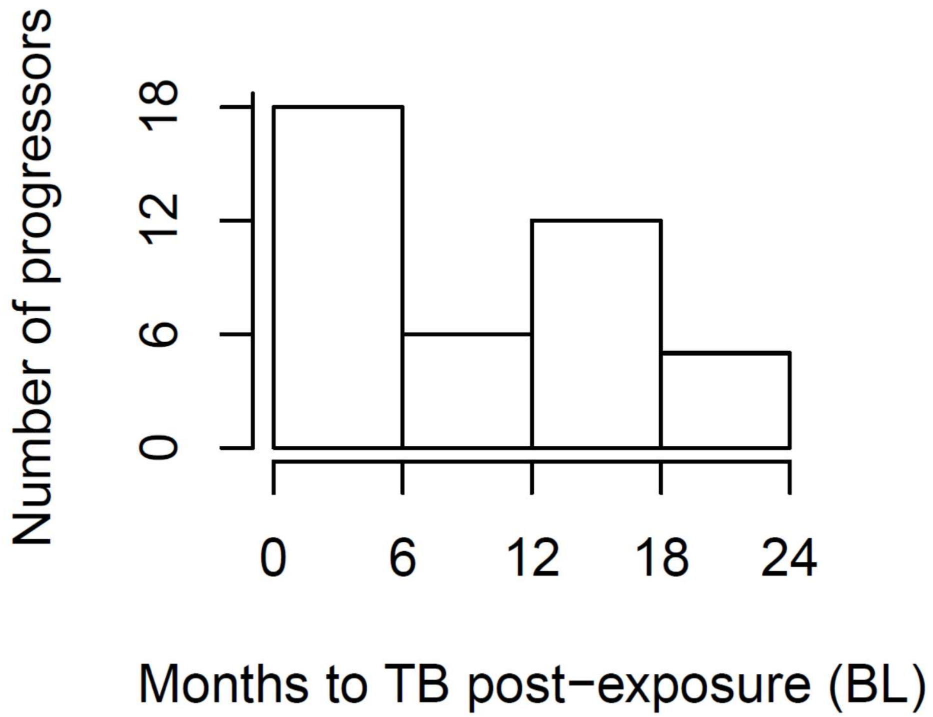

Figure S3: Distribution of times to TB for HHCs. Histogram of how long each progressor took to develop active TB from baseline/enrollment.

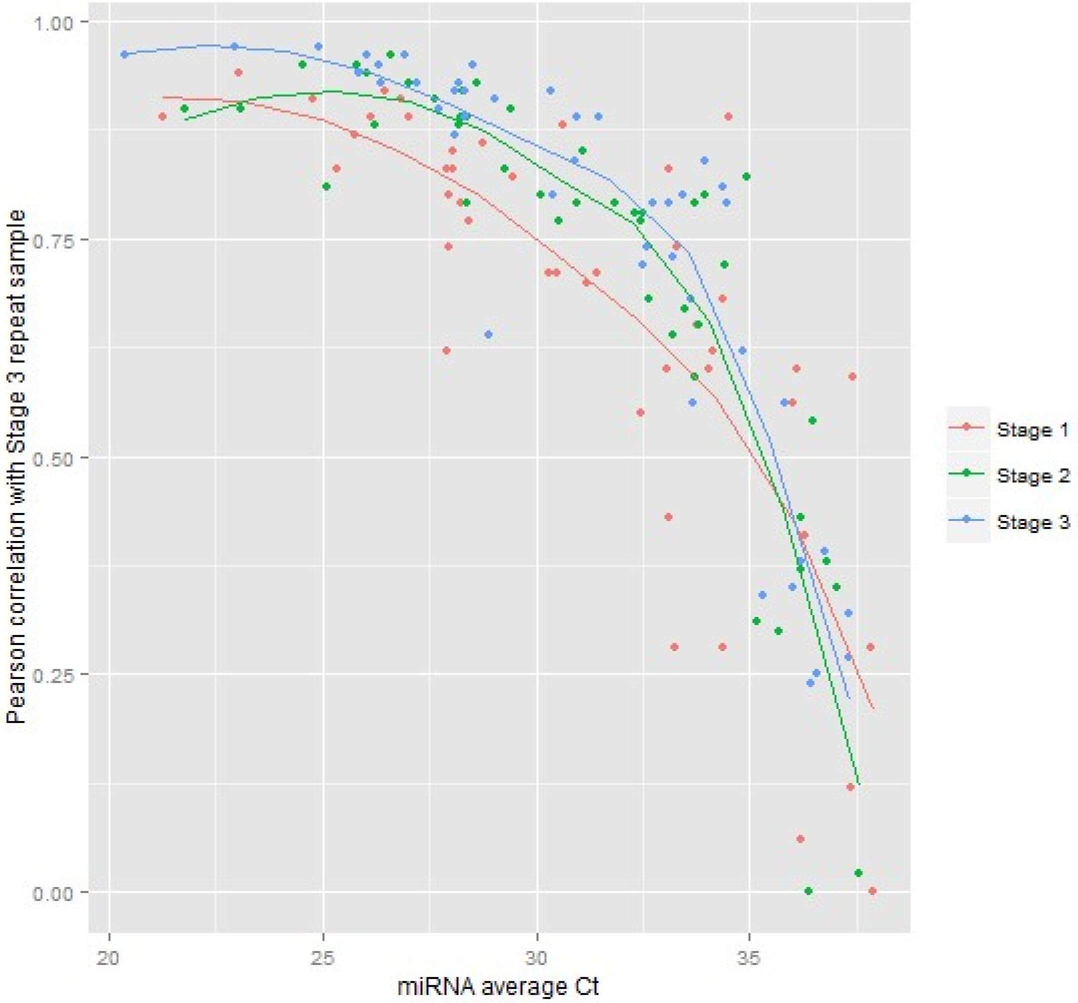

Figure S4: Technical replicability of miRNAs. Correlations between technical replicate miRNAs measured independently during the initial downsampling based on 40 training samples (Stage 1); the entire training set (Stage 2); and the first replicate of the entire training+test set (Stage 3) vs. the second replicate from the training+test sets show the influence of miRNA average Ct (x-axis) on replicability (y-axis: Pearson correlation between technical replicates).
